# (μ-2′,6′-Dicarboxy­biphenyl-2,6-dicar­boxylato)bis[(1,10-phenanthroline)silver(I)]

**DOI:** 10.1107/S1600536810037700

**Published:** 2010-09-30

**Authors:** Lin Cheng, Jian-Quan Wang, Li-Min Zhang

**Affiliations:** a Department of Chemistry and Chemical Engineering, Southeast University, Nanjing, 211189, People’s Republic of China

## Abstract

In the dimeric title complex, [Ag_2_(C_16_H_8_O_8_)(C_12_H_8_N_2_)_2_] or [Ag_2_(H_2_bta)(phen)_2_] (H_4_bta = biphenyl-2,2′,6,6′-tetra­carb­ox­y­lic acid, phen = 1,10-phenanthroline), each Ag(I) ion displays an approximatively planar-trigonal geometry, being surrounded by one chelating phen ligand and one carboxyl­ate O atom from an H_2_bta ligand. Owing to the the presence of crystallographic twofold rotation axes, the four C atoms bisecting the H~2~bta ligand are located on a special position. Each H_2_bta ligand acts as a bis-monodentate ligand, ligating two Ag(I) ions into a dimeric compound. Inter­molecular O—H⋯O inter­actions are observed in the crystal structure.

## Related literature

The self-assembled construction of coordination polymers is of current inter­est in the field of supra­molecular chemistry and crystal engineering owing to their potential applications as functional materials, as well as their intriguing variety of architectures and mol­ecular topologies, see: Braga *et al.* (1998 ▶); Yaghi *et al.* (1998 ▶). For related structures, see: Huang *et al.* (2007 ▶); Suh *et al.* (2006 ▶).
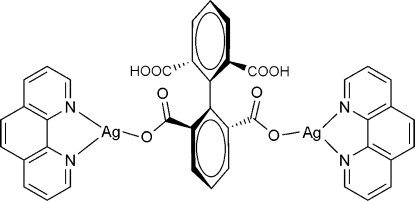


## Experimental

### 

#### Crystal data

[Ag_2_(C_16_H_8_O_8_)(C_12_H_8_N_2_)_2_]
*M* *_r_* = 904.37
Monoclinic, 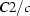

*a* = 11.7633 (17) Å
*b* = 11.5730 (18) Å
*c* = 24.884 (4) Å
β = 92.397 (2)°
*V* = 3384.7 (9) Å^3^
*Z* = 4
Mo *K*α radiation
μ = 1.22 mm^−1^
*T* = 293 K
0.20 × 0.18 × 0.15 mm

#### Data collection

Bruker APEX CCD diffractometer
Absorption correction: multi-scan (*SADABS*; Sheldrick, 2000 ▶) *T*_min_ = 0.792, *T*_max_ = 0.838
8881 measured reflections
3300 independent reflections
1843 reflections with *I* > 2σ(*I*)
*R*_int_ = 0.041

#### Refinement

*R*[*F* ^2^ > 2σ(*F* ^2^)] = 0.057
*wR*(*F* ^2^) = 0.178
*S* = 1.04
3300 reflections
246 parameters
H-atom parameters constrained
Δρ_max_ = 1.59 e Å^−3^
Δρ_min_ = −0.97 e Å^−3^

### 

Data collection: *SMART* (Bruker, 2000 ▶); cell refinement: *SAINT* (Bruker, 2000 ▶); data reduction: *SAINT*; program(s) used to solve structure: *SHELXTL* (Sheldrick, 2008 ▶); program(s) used to refine structure: *SHELXTL*; molecular graphics: *SHELXTL*; software used to prepare material for publication: *SHELXTL*.

## Supplementary Material

Crystal structure: contains datablocks I, global. DOI: 10.1107/S1600536810037700/kp2272sup1.cif

Structure factors: contains datablocks I. DOI: 10.1107/S1600536810037700/kp2272Isup2.hkl

Additional supplementary materials:  crystallographic information; 3D view; checkCIF report

## Figures and Tables

**Table 1:** Selected bond lengths (Å)

Ag1—O1	2.141 (4)
Ag1—N2	2.220 (5)
Ag1—N1	2.350 (6)

**Table 2:** Hydrogen-bond geometry (Å, °)

*D*—H⋯*A*	*D*—H	H⋯*A*	*D*⋯*A*	*D*—H⋯*A*
O4—H4*A*⋯O2^i^	0.85	1.71	2.564 (7)	179

Symmetry code: (i) 
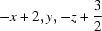
.

**Fig. 1.**
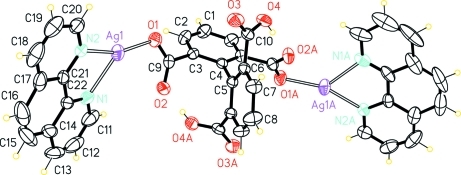
Structure of the title compound with 30% thermal ellipsoids. Symmetry code: A: 2 - x, y, 3/2 - z.

## supplementary crystallographic information

### Comment

The self-assembled construction of coordination polymers is of current interest in the field of supramolecular chemistry and crystal engineering owing to their potential applications as functional materials, as well as their intriguing variety of architectures and molecular topologies (Braga *et al.* 1998; Yaghi *et al.* 1998). Polycarboxylate ligands,such as 1,2-benzenedicarboxylate, 1,3,5-benzenetricarboxylate and 1,2,4,5-benzenetetracarboxylate, have been extensively employed in the preparation of such coordination polymers in possession of multidimensional networks and interesting properties. However, the biphenyl-2,2',6,6'-tetracarboxylic acid (H_4_bta) ligand, as a member of multidentate O-donor ligands, is rarely used (Huang *et al.* 2007; Suh *et al.* 2006). Herein, we synthesis a new dimeric complex, Ag_2_(H_2_bta)(phen)_2_ (H_4_bta = biphenyl-2,2',6,6'-tetracarboxylic acid, phen = 1,10-phenanthroline) with H_4_bta ligand.

The asymmetric unit of the title compound, contains a Ag(I) cation, half of a H_2_bta ligand and a chelating phen. In the compound, each Ag(I) ion displays a approximatively planar trigonal geometry, being surrounde by one chelating phen ligand and one carboxylate oxygen atom, coming from a H_2_bta ligand. Each H_2_bta acts as a bis-monodentate ligand to ligate two Ag(I) ions into a dimeric compound. Interestingly, only the two carboxylates in one benzene ring of each H_2_bta ligand are deprotoned and coordinated to Ag(I) ions, while the other two carboxylates in the other benzene ring are undeprotoned and free. The Ag···Ag distance in the dimer is 10.61 (1) Å. It is noted that the angle of two benzene rings in a H_2_bta ligand is 70.52 (4)°.

### Experimental

A mixture of H_4_bta (0.066 g, 0.2 mmol), AgNO_3_ (0.068 g, 0.4 mmol), phen (0.072 g, 0.4 mmol) and H_2_O (10 ml) were heated in a 25-ml Teflon-lined vessel at 180 ° for 3 days, followed by slow cooling (5 ° h^-1^) to room temperature. After filtration and washing with H_2_O, colourless block crystals were collected and dried in air (0.054 g, yield *ca* 15% based on H_4_bta).

### Refinement

The H atoms of carboxylates were located in a difference map and was refined with the restraint of O—H = 0.85 Å. Other H atoms were positioned geometrically and refined using a riding model with C—H = 0.93 Å, and with *U*_iso_(H) = 1.2 *U*_iso_(C).

### Crystal data

**Table:**

[Ag_2_(C_16_H_8_O_8_)(C_12_H_8_N_2_)_2_]	*F*(000) = 1800
*M**_r_* = 904.37	*D*_x_ = 1.775 Mg m^−^^3^
Monoclinic, *C*2/*c*	Mo *K*α radiation, λ = 0.71073 Å
Hall symbol: -C 2yc	Cell parameters from 785 reflections
*a* = 11.7633 (17) Å	θ = 2.4–28.0°
*b* = 11.5730 (18) Å	µ = 1.22 mm^−^^1^
*c* = 24.884 (4) Å	*T* = 293 K
β = 92.397 (2)°	Block, colourless
*V* = 3384.7 (9) Å^3^	0.20 × 0.18 × 0.15 mm
*Z* = 4	

### Data collection

**Table:**

Bruker APEX CCD diffractometer	3300 independent reflections
Radiation source: fine-focus sealed tube	1843 reflections with *I* > 2σ(*I*)
graphite	*R*_int_ = 0.041
phi and ω scan	θ_max_ = 26.0°, θ_min_ = 2.5°
Absorption correction: multi-scan (*SADABS*; Sheldrick, 2000)	*h* = −14→11
*T*_min_ = 0.792, *T*_max_ = 0.838	*k* = −14→13
8881 measured reflections	*l* = −30→24

### Refinement

**Table:**

Refinement on *F*^2^	Primary atom site location: structure-invariant direct methods
Least-squares matrix: full	Secondary atom site location: difference Fourier map
*R*[*F*^2^ > 2σ(*F*^2^)] = 0.057	Hydrogen site location: inferred from neighbouring sites
*wR*(*F*^2^) = 0.178	H-atom parameters constrained
*S* = 1.04	*w* = 1/[σ^2^(*F*_o_^2^) + (0.0855*P*)^2^ + 3.1208*P*] where *P* = (*F*_o_^2^ + 2*F*_c_^2^)/3
3300 reflections	(Δ/σ)_max_ = 0.001
246 parameters	Δρ_max_ = 1.59 e Å^−^^3^
0 restraints	Δρ_min_ = −0.97 e Å^−^^3^

### Special details

**Table:**

Geometry. All e.s.d.'s (except the e.s.d. in the dihedral angle between two l.s. planes) are estimated using the full covariance matrix. The cell e.s.d.'s are taken into account individually in the estimation of e.s.d.'s in distances, angles and torsion angles; correlations between e.s.d.'s in cell parameters are only used when they are defined by crystal symmetry. An approximate (isotropic) treatment of cell e.s.d.'s is used for estimating e.s.d.'s involving l.s. planes.
Refinement. Refinement of *F*^2^ against ALL reflections. The weighted *R*-factor *wR* and goodness of fit *S* are based on *F*^2^, conventional *R*-factors *R* are based on *F*, with *F* set to zero for negative *F*^2^. The threshold expression of *F*^2^ > σ(*F*^2^) is used only for calculating *R*-factors(gt) *etc*. and is not relevant to the choice of reflections for refinement. *R*-factors based on *F*^2^ are statistically about twice as large as those based on *F*, and *R*- factors based on ALL data will be even larger.

### Fractional atomic coordinates and isotropic or equivalent isotropic displacement parameters (Å^2^)

**Table:**

	*x*	*y*	*z*	*U*_iso_*/*U*_eq_	
Ag1	0.81077 (5)	0.01234 (5)	0.55273 (2)	0.0794 (3)	
C1	1.0000	−0.2818 (8)	0.7500	0.070 (3)	
H1A	1.0000	−0.3622	0.7500	0.085*	
C2	0.9466 (6)	−0.2224 (5)	0.7086 (3)	0.0664 (18)	
H2A	0.9108	−0.2627	0.6803	0.080*	
C3	0.9455 (5)	−0.1024 (5)	0.7085 (2)	0.0484 (13)	
C4	1.0000	−0.0408 (7)	0.7500	0.0429 (17)	
C5	1.0000	0.0907 (7)	0.7500	0.0461 (18)	
C6	1.0860 (4)	0.1536 (5)	0.7259 (2)	0.0473 (13)	
C7	1.0835 (5)	0.2719 (6)	0.7253 (3)	0.0659 (17)	
H7A	1.1395	0.3122	0.7077	0.079*	
C8	1.0000	0.3321 (8)	0.7500	0.083 (3)	
H8A	1.0000	0.4125	0.7500	0.099*	
C9	0.8803 (6)	−0.0437 (5)	0.6624 (3)	0.0556 (15)	
C10	1.1845 (5)	0.0952 (6)	0.6995 (3)	0.0559 (16)	
C11	0.5481 (9)	0.0452 (9)	0.5938 (4)	0.108 (3)	
H11A	0.5717	−0.0003	0.6229	0.130*	
C12	0.4429 (11)	0.0925 (13)	0.5920 (6)	0.145 (6)	
H12A	0.3943	0.0762	0.6196	0.173*	
C13	0.4064 (9)	0.1619 (13)	0.5520 (7)	0.148 (7)	
H13A	0.3345	0.1953	0.5524	0.178*	
C14	0.4794 (7)	0.1846 (9)	0.5079 (5)	0.107 (3)	
C15	0.4506 (9)	0.2534 (11)	0.4630 (8)	0.153 (7)	
H15A	0.3805	0.2907	0.4615	0.183*	
C16	0.5196 (12)	0.2673 (8)	0.4225 (5)	0.132 (5)	
H16A	0.4964	0.3137	0.3936	0.158*	
C17	0.6320 (8)	0.2111 (6)	0.4224 (4)	0.085 (2)	
C18	0.7075 (11)	0.2189 (8)	0.3815 (4)	0.111 (3)	
H18A	0.6891	0.2618	0.3507	0.133*	
C19	0.8068 (10)	0.1640 (8)	0.3867 (3)	0.099 (3)	
H19A	0.8572	0.1669	0.3590	0.118*	
C20	0.8361 (6)	0.1025 (6)	0.4333 (3)	0.0734 (19)	
H20A	0.9071	0.0671	0.4363	0.088*	
C21	0.6640 (5)	0.1444 (5)	0.4685 (3)	0.0574 (15)	
C22	0.5854 (5)	0.1301 (6)	0.5117 (3)	0.071 (2)	
N1	0.6176 (5)	0.0637 (6)	0.5541 (2)	0.0738 (16)	
N2	0.7670 (4)	0.0925 (4)	0.47350 (19)	0.0550 (12)	
O1	0.9113 (4)	−0.0678 (4)	0.61601 (18)	0.0740 (13)	
O2	0.8007 (4)	0.0204 (4)	0.67257 (19)	0.0720 (13)	
O3	1.2028 (4)	0.1158 (4)	0.65313 (19)	0.0761 (13)	
O4	1.2503 (4)	0.0277 (4)	0.7283 (2)	0.0683 (12)	
H4A	1.2340	0.0253	0.7612	0.082*	

### Atomic displacement parameters (Å^2^)

**Table:**

	*U*^11^	*U*^22^	*U*^33^	*U*^12^	*U*^13^	*U*^23^
Ag1	0.0838 (5)	0.0943 (5)	0.0584 (4)	0.0106 (3)	−0.0170 (3)	−0.0002 (3)
C1	0.095 (7)	0.047 (5)	0.070 (7)	0.000	0.005 (6)	0.000
C2	0.085 (5)	0.057 (4)	0.057 (4)	−0.012 (3)	0.001 (4)	−0.007 (3)
C3	0.051 (3)	0.046 (3)	0.048 (3)	0.002 (2)	0.001 (3)	−0.005 (3)
C4	0.037 (4)	0.049 (5)	0.043 (4)	0.000	0.005 (3)	0.000
C5	0.045 (4)	0.050 (5)	0.043 (4)	0.000	0.000 (4)	0.000
C6	0.043 (3)	0.052 (4)	0.047 (3)	−0.006 (3)	0.005 (2)	0.001 (3)
C7	0.066 (4)	0.058 (4)	0.075 (5)	−0.009 (3)	0.022 (3)	0.007 (3)
C8	0.103 (8)	0.045 (5)	0.103 (8)	0.000	0.029 (7)	0.000
C9	0.053 (4)	0.060 (4)	0.053 (4)	−0.007 (3)	−0.007 (3)	−0.004 (3)
C10	0.039 (3)	0.068 (4)	0.061 (4)	−0.007 (3)	0.010 (3)	−0.004 (3)
C11	0.091 (7)	0.133 (7)	0.105 (7)	−0.049 (6)	0.042 (6)	−0.058 (6)
C12	0.091 (10)	0.180 (15)	0.167 (13)	−0.055 (9)	0.050 (8)	−0.122 (11)
C13	0.045 (6)	0.167 (13)	0.233 (17)	−0.008 (6)	0.023 (9)	−0.141 (12)
C14	0.064 (6)	0.107 (7)	0.149 (9)	0.019 (5)	−0.018 (6)	−0.076 (7)
C15	0.069 (7)	0.115 (9)	0.27 (2)	0.030 (6)	−0.071 (9)	−0.063 (12)
C16	0.144 (11)	0.074 (6)	0.169 (12)	0.022 (7)	−0.092 (9)	−0.002 (7)
C17	0.112 (6)	0.054 (4)	0.084 (6)	−0.001 (4)	−0.039 (5)	−0.012 (4)
C18	0.200 (12)	0.072 (6)	0.057 (5)	−0.018 (6)	−0.022 (7)	0.007 (4)
C19	0.152 (9)	0.084 (6)	0.062 (6)	−0.029 (6)	0.019 (6)	−0.005 (5)
C20	0.073 (5)	0.086 (5)	0.061 (5)	−0.011 (4)	0.007 (4)	−0.015 (4)
C21	0.059 (4)	0.053 (4)	0.059 (4)	0.003 (3)	−0.016 (3)	−0.012 (3)
C22	0.044 (4)	0.077 (5)	0.091 (6)	0.001 (3)	−0.007 (4)	−0.043 (4)
N1	0.061 (4)	0.098 (5)	0.063 (4)	−0.014 (3)	0.015 (3)	−0.027 (3)
N2	0.057 (3)	0.062 (3)	0.046 (3)	0.002 (2)	0.001 (2)	−0.011 (2)
O1	0.075 (3)	0.098 (4)	0.048 (3)	0.009 (3)	−0.004 (2)	0.001 (2)
O2	0.065 (3)	0.088 (3)	0.062 (3)	0.021 (2)	−0.009 (2)	−0.003 (2)
O3	0.067 (3)	0.102 (4)	0.061 (3)	0.003 (2)	0.024 (2)	0.005 (3)
O4	0.049 (2)	0.085 (3)	0.071 (3)	0.011 (2)	0.013 (2)	0.006 (2)

### Geometric parameters (Å, °)

**Table:**

Ag1—O1	2.141 (4)	C11—C12	1.352 (15)
Ag1—N2	2.220 (5)	C11—H11A	0.9300
Ag1—N1	2.350 (6)	C12—C13	1.337 (18)
C1—C2	1.369 (8)	C12—H12A	0.9300
C1—C2^i^	1.369 (8)	C13—C14	1.445 (17)
C1—H1A	0.9300	C13—H13A	0.9300
C2—C3	1.389 (8)	C14—C22	1.397 (11)
C2—H2A	0.9300	C14—C15	1.401 (18)
C3—C4	1.390 (7)	C15—C16	1.329 (17)
C3—C9	1.514 (8)	C15—H15A	0.9300
C4—C3^i^	1.390 (7)	C16—C17	1.474 (14)
C4—C5	1.521 (11)	C16—H16A	0.9300
C5—C6^i^	1.402 (6)	C17—C18	1.383 (12)
C5—C6	1.402 (6)	C17—C21	1.419 (10)
C6—C7	1.370 (8)	C18—C19	1.332 (13)
C6—C10	1.515 (8)	C18—H18A	0.9300
C7—C8	1.371 (8)	C19—C20	1.391 (11)
C7—H7A	0.9300	C19—H19A	0.9300
C8—C7^i^	1.371 (8)	C20—N2	1.320 (8)
C8—H8A	0.9300	C20—H20A	0.9300
C9—O2	1.230 (8)	C21—N2	1.353 (7)
C9—O1	1.255 (7)	C21—C22	1.457 (10)
C10—O3	1.207 (7)	C22—N1	1.346 (9)
C10—O4	1.295 (7)	O4—H4A	0.8492
C11—N1	1.326 (9)		
			
O1—Ag1—N2	159.20 (18)	C12—C13—C14	119.6 (12)
O1—Ag1—N1	127.1 (2)	C12—C13—H13A	120.2
N2—Ag1—N1	73.7 (2)	C14—C13—H13A	120.2
C2—C1—C2^i^	119.7 (9)	C22—C14—C15	119.8 (11)
C2—C1—H1A	120.1	C22—C14—C13	115.0 (12)
C2^i^—C1—H1A	120.1	C15—C14—C13	125.2 (11)
C1—C2—C3	120.5 (6)	C16—C15—C14	122.5 (11)
C1—C2—H2A	119.8	C16—C15—H15A	118.7
C3—C2—H2A	119.8	C14—C15—H15A	118.7
C2—C3—C4	120.5 (6)	C15—C16—C17	121.6 (11)
C2—C3—C9	117.0 (5)	C15—C16—H16A	119.2
C4—C3—C9	122.5 (5)	C17—C16—H16A	119.2
C3—C4—C3^i^	118.3 (7)	C18—C17—C21	118.3 (8)
C3—C4—C5	120.9 (4)	C18—C17—C16	125.2 (10)
C3^i^—C4—C5	120.9 (4)	C21—C17—C16	116.5 (9)
C6^i^—C5—C6	117.4 (7)	C19—C18—C17	119.0 (8)
C6^i^—C5—C4	121.3 (3)	C19—C18—H18A	120.5
C6—C5—C4	121.3 (3)	C17—C18—H18A	120.5
C7—C6—C5	120.6 (5)	C18—C19—C20	120.8 (8)
C7—C6—C10	117.2 (5)	C18—C19—H19A	119.6
C5—C6—C10	122.2 (5)	C20—C19—H19A	119.6
C6—C7—C8	121.2 (6)	N2—C20—C19	122.5 (8)
C6—C7—H7A	119.4	N2—C20—H20A	118.7
C8—C7—H7A	119.4	C19—C20—H20A	118.7
C7^i^—C8—C7	118.9 (9)	N2—C21—C17	121.5 (7)
C7^i^—C8—H8A	120.5	N2—C21—C22	118.4 (6)
C7—C8—H8A	120.5	C17—C21—C22	120.2 (7)
O2—C9—O1	125.2 (6)	N1—C22—C14	122.2 (9)
O2—C9—C3	118.7 (6)	N1—C22—C21	118.4 (6)
O1—C9—C3	116.1 (6)	C14—C22—C21	119.4 (9)
O3—C10—O4	121.5 (5)	C11—N1—C22	120.8 (8)
O3—C10—C6	119.8 (6)	C11—N1—Ag1	126.7 (7)
O4—C10—C6	118.6 (6)	C22—N1—Ag1	112.0 (4)
N1—C11—C12	120.2 (12)	C20—N2—C21	117.9 (6)
N1—C11—H11A	119.9	C20—N2—Ag1	125.6 (5)
C12—C11—H11A	119.9	C21—N2—Ag1	116.2 (4)
C13—C12—C11	122.1 (13)	C9—O1—Ag1	114.0 (4)
C13—C12—H12A	118.9	C10—O4—H4A	113.5
C11—C12—H12A	118.9		

Symmetry codes: (i) −*x*+2, *y*, −*z*+3/2.

### Hydrogen-bond geometry (Å, °)

**Table:**

*D*—H···*A*	*D*—H	H···*A*	*D*···*A*	*D*—H···*A*
O4—H4A···O2^i^	0.85	1.71	2.564 (7)	179

Symmetry codes: (i) −*x*+2, *y*, −*z*+3/2.
